# Knowing how, arguing why: nurse anaesthetists’ experiences of nursing when caring for the surgical patient

**DOI:** 10.1186/s12912-025-02752-3

**Published:** 2025-02-07

**Authors:** Aina L. Bjerkeli Lekens, Sigrunn Drageset, Britt Sætre Hansen

**Affiliations:** 1 Faculty of Health, University of Stavanger, Stavanger, Norway; 2 Faculty of Health and Social Sciences, Western Norway University of Applied Sciences, Bergen, Norway

**Keywords:** Nurse anaesthetist, Nursing care, Surgical patient, Competency

## Abstract

**Background:**

Patients undergoing surgery and anaesthesia are in a vulnerable situation, requiring advanced treatment and care. Nurse anaesthetists are specially trained to meet their needs when administering and monitoring anaesthesia, ensuring patient safety and comfort throughout the entire perioperative care. However, there is a growing concern that efficiency requirements might take precedence over humanistic care when having to prioritize. Most people have a limited understanding of the extended role of nurse anaesthetists in maintaining quality and safety in anaesthesia services. This study aimed to explore how nurse anaesthetists describe their practice as being nursing when caring for surgical patients.

**Methods:**

We applied a qualitative inductive design. Twenty nurse anaesthetists working in small or large hospitals in different regions of Norway were recruited. Semi-structured individual interviews were carried out. Data were analysed using qualitative content analysis. This study was conducted and reported in accordance with the Consolidated Criteria for Reporting Qualitative Research Checklist.

**Results:**

We identified three categories and eight subcategories. The category *Continuously* a*ttending to physical and psychological needs* involved reading the patient and responding accordingly and the ethical and moral obligation to maintain patients’ well-being and dignity. The category *Providing a concerned presence* involved the importance of the nursing process and nurse anaesthetists’ self-confidence in their professional abilities and readiness to act when caring for the surgical patient. The category *Aspiring towards excellence* involved expectations towards nurse anaesthetists as professionals who display professional courage and undertake professional development through continuous learning.

**Conclusion:**

Nurse anaesthetists experience themselves as being highly qualified to address the needs of surgical patients. Their nursing background and extensive training have prepared them to know how and argue why. Knowledge from this study could be expected to add to the field of describing and understanding nurse anaesthetists’ practice in the perioperative context and support supervision in postgraduate education.

## Background

In 2015, the World Health Assembly recognized surgery and anaesthesia as vital components of universal health coverage, aiming to ensure global access to these services by 2030 [1]. In 2021, the International Council of Nurses (ICN) followed up by releasing guidelines on advanced practice nursing, and nurse anaesthetists (NAs) specifically, to clarify their practice, emphasizing the crucial role of nurses in achieving this goal [2]. According to the International Federation of Nurse Anaesthetists (IFNA) recommendations, an NA is an advanced practice nurse (APN) who has completed a basic generalist nursing education programme and a recognized anaesthesia educational programme at the postgraduate level, providing an expanded level of care beyond the practice of a generalist nurse [2]. However, there is great variation in the identification and context of NAs across different countries, including education, regulation, and healthcare practice [2, 3]. The IFNA Education Committee strongly recommends that applicants for NA programs must have completed a basic nursing education program of at least 36 months and have at least one year of nursing experience, preferably in an acute care setting. Further, the committee endorses an 18 to 24-month educational program, recommending that at least 50% of it be dedicated to clinical learning experiences with direct patient care [2]. The ICN recommends that the minimum standard for educational preparations should be at a master’s level, while also acknowledging that for many countries this is aspirational due to limited resources. Therefore, graduates are provided a certificate, diploma, or degree appropriate to the education level and legislation to provide nurse anaesthesia services in each respective country. The Norwegian educational system is compliant with these recommendations. In general, NAs administer and monitor anaesthesia, ensuring patient safety and comfort throughout the entire perioperative care process. Their responsibilities include preparing equipment, administering drugs, managing pain and monitoring vital signs [2]. In low-income countries, NAs are often the sole providers of anaesthesia services. In high-income countries, such as the Scandinavian countries, Switzerland and the USA, NAs commonly work together with or under indirect supervision of an anaesthesiologist [3].

The perioperative environment is characterised by its complexity, advanced technology, high pace and high risk. Patients undergoing surgery and anaesthesia face inherent uncertainty and risk, placing them in a vulnerable situation. From a patient perspective, this is often described as placing your life in the hands of others [4–5]. For the patient, there is a qualitative difference between the perception of feeling safe and actually being safe [6]. Patient experience with healthcare systems in general is positively linked to clinical patient safety and quality, suggesting that patient experiences should be taken into account when designing healthcare systems [7].

According to Kim [8], nursing practice is intentional, thought-out and goal-directed in response to patients’ specific needs, meaning that the context of application is always important. Nursing is also a caring practice, encompassing humanistic values such as empathy, trust, dignity and mutuality [9, 10]. In anaesthesia practice specifically, there are particular underlying ontological concerns for nursing that call for an appropriate nursing response, such as pain, postoperative nausea and vomiting, anxiety, postoperative cognitive dysfunction, despair and hope [11, 12]. New knowledge and technological innovations offer great advances within surgery and anaesthesiology. However, there is also a risk of a dehumanizing influence linked to workforce challenges and efficiency requirements [13, 14]. There is growing concern that biomedical knowledge and financial concerns will take precedence over humanistic care when having to prioritize [15], leading to a rationing of nursing care and for certain aspects of nursing care to be rendered invisible and even devalued [16, 17].

Over the years, some practitioners and researchers have expressed their concerns about a dilution of the original discipline of nursing when borrowing and integrating theories and concepts from other disciplines. APNs especially, are often criticized as being “junior doctors” and not practicing nursing, as they tend to relate closely to a medical model [18, 19]. As anaesthesia nursing commonly takes place in a restricted area, most people, including other nurses, have a limited understanding of the extended role of NAs [18]. This is reinforced as most of their patients are under general anaesthesia and therefore unaware of their surroundings.

Nursing practice requires technical skills, but good practice goes beyond such skills and can be seen as a defined activity based on a shared culture and tradition [9]. Given this background, the present study aimed to explore how NAs describe their practice as being nursing when caring for surgical patients in need of anaesthesia services.

## Methods

This study employed a qualitative inductive design, which allowed us to explore and analyse experiences from individuals to gain a deeper understanding of the phenomenon, moving from the particular into a greater whole [20].

### Setting and participants

Participants were recruited via social media. Purposive sampling was considered most suitable. The inclusion criterion was being currently employed as an NA in Norway.

### Data collection

Individual interviews were conducted digitally via Zoom and audio recorded by the first author between February and April 2023, lasting from 23 to 76 min, with an average of 44 min. A semi-structured interview guide was used (Table 1). The interview guide was developed based on the research team’s extensive experience in the field, two with a previous background as NAs and one as an intensive care nurse. We aimed to formulate questions that would elicit rich, detailed responses from participants. Follow-up questions were used to obtain deeper insights where appropriate. The first interview was piloted and included in the data as no revisions of the interview guide were necessary.

**Table 1:** Semi-structured interview guide

1. What is nursing to you in your daily practice of anaesthesia nursing? How and why?
2. What do you consider central in the direct meeting with the patient? How and why?
3. How do you involve the patient in the treatment? And next of kin?
4. Describe, how do you use yourself when dealing with patients?
5. Which skills do you believe a nurse anaesthetist should have when caring for the patient, and why?
6. When time is restricted, how do you prioritize, and why?

### Data analysis

The interviews were immediately transcribed verbatim by the first author. Data were analysed using qualitative content analysis as described by Graneheim, Lindgren and Lundman [21, 22]. The first and last author read the transcripts repeatedly to develop a sense of the material and overall domains [23]. The first author then separated the text into meaning units that were condensed and coded using NVIVO [24]. Transcripts were read inductively, and then similar codes were grouped, interpretated and abstracted into categories and subcategories. All three authors were involved in the final analytical process. This was a back-and-forth process where themes and concepts were revised through discussion, providing us with new perspectives.

### Ethical considerations

The study was performed in accordance with the Declaration of Helsinki and formally approved by the Norwegian Agency for Shared Services in Education and Research (reference number 425727). All participants received written and oral information about the study and gave their written consent prior to the interviews. The researcher conducting the interviews had limited knowledge of the workplace of each participant, enabling them to speak freely about the topic. All recordings and transcripts were encrypted and stored in a secure place available only to the researchers.

## Results

Twenty NAs aged 27–65 years (five males and fifteen females) participated in individual interviews. The mean age was 47.4 years, with an average of 15.3 years of experience as an NA. The type of hospital for their current employment was well distributed, with ten working at a university hospital, three at a regional hospital and seven at a local hospital.

Our analysis revealed that the nursing in nurse anaesthesia is a refinement and continuous transformation of basic nursing skills and knowledge into advanced practice nursing in the context of anaesthesia and perioperative care. This transition depends on a commitment to continuous education and developing skills and knowledge through experience, with the aim of providing excellent quality of care. The analysis resulted in three categories: *Attending to physical and psychological needs*, *Providing a concerned presence* and *Aspiring towards excellence*, with eight subcategories as shown in Table 2.

**Table 2:** Overview of the identified categories and subcategories

Categories	Subcategories
Continuously attending to physical and psychological needs	Reading the patient and responding accordingly Ethical standards grounded in nursing as a profession
Providing a concerned presence	Engaging genuinely Knowing oneself Combining advanced knowledge with experience
Aspiring towards excellence	Living up to professional expectations Exercising professional courage Continuously learning

### Continuously attending to physical and psychological needs

The NAs highlighted the importance of their previous experience as generalist nurses when caring for patients as a whole, as they attended to both physical and psychological needs. This involved reading each patient, but it was also regarded as an ethical and moral obligation to maintain the patients’ well-being and dignity. One NA summarized this as follows:

*“I often think about this as head-heart-hand. You must have the theory in your head. You must have care in your heart. The ethics and the moral. And then you have to have the skills in your hands.”* (NA14).

### Reading the patient and responding accordingly

Taking care of the patients’ basic needs was described as the core of nursing in anaesthesia, such as maintaining physical equilibrium, relieving pain and nausea, regulating body temperature and positioning the patient on the surgical table to prevent neurological and tissue damage. Previous experience as a nurse had prepared them by sharpening the skills necessary to solve more advanced situations, especially in being able to observe and compile data systematically from various sources, creating a meaningful understanding of complex situations. As a result, they now understood *why* and were able to argue their case.

*“When you’re becoming a nurse anaesthetist*,* those skills are further developed. They get sharpened. I’m still very much a nurse*,* but that’s what observation skills are all about. One must be able to observe.”* (NA20).

The relational competence and ability to read a fellow human being in crisis was rooted in their basic education and identity as a nurse, whereas handling advanced medical and technical equipment was refined through further education and training. The NAs also came with a clear warning not to get lost in technology, but to maintain relational competency. Many reported a noticeable change in anaesthesia nursing over the past decades, moving from a task-oriented profession to one focusing more on non-technical skills.

*“But you know that your background as a nurse is there. Then you can use that expertise in the argumentation. And then it stands its ground.”* (NA19).

Interestingly, some areas of nursing were described as invisible care, as patients are often unconscious and therefore unaware of the measures taken during sleep. But nursing care was also regarded as being invisible to the patients in the sense of not being aware of the risks involved in the first place. Some pointed out how the systems available for documenting care and treatment in anaesthesia situations are mainly designed to cover medical treatment and technical procedures, rather than nursing care, rendering it also invisible for the records.

*“There is something about these tools. They are not necessarily suitable for documenting actual nursing itself.”* (NA15).

This made it more difficult to get a hold of the patient’s previous experiences or track down previous measures taken to provide the patient with individual care. Therefore, it was important to use the short time available in the initial meeting with the patient to capture the patient’s individual needs and concerns. They wanted patients to experience care in terms of both physical and psychological well-being.

### Ethical standards grounded in nursing as a profession

All participants emphasized their moral obligation to maintain professional standards and promote best practice. Their basic training as a nurse had provided them with a foundation for the value-based care necessary for interacting with patients and their relatives in vulnerable situations. It was also regarded as imperative for developing clinical reasoning and assessment skills. This made them confident to engage in negotiations about what is best for each individual patient. They would contribute substantively to discussions, where identifying the right moment to address patients’ needs was crucial. Sometimes dilemmas arose between safeguarding dignity and ensuring patient safety.

*“Many focus on the new technology*,* or the medical explanations. Then we might pull in a slightly different direction in relation to observations and how*,* yes*,* practical implementation*,* and what might suit this and that patient best. There may not always be a gold standard that suits everyone.”* (NA20).

It was pointed out that nursing is a distinct profession with its own ethical standards and guidelines. Having previous experiences of working in teams with other professions enabled them to take on multiple perspectives. This led to an increased awareness of the hospital culture and being part of a larger team where everyone complements each other. The aim was always to ensure that the patient was left with a feeling of being cared for as a whole.

*“The nursing component is a very complementary and independent subject within what we do. We have our own ethical guidelines*,* our own standards*,* our own assessments. […] It is a privilege that we can be a doctor and a nurse when needed*,* and that we can be two nurses when needed.”* (NA20).

### Providing a concerned presence

The NAs were aware of their accountability when they were involved in perioperative anaesthesia care. They emphasized the importance of the nursing process, understood as continuous observation, action, evaluation and staying ahead of every situation. Knowing oneself and having confidence in one’s own ability to exercise substantial clinical judgement allowed them to engage genuinely in building a trustful relationship with the patient, thereby providing a concerned presence and readiness to act.

### Engaging genuinely

The participants recognized that their patients were in a vulnerable situation and emphasized the importance of engaging genuinely with the patient. Within a few minutes, they would form an impression of the patient and adjust to the situation accordingly. The aim was to create a trusting relationship while carrying out practical tasks, illustrating clearly that they were in control and relaxed about the situation. They used themselves as a tool, for example, through eye contact, physical touch and the use of humour. It was important to see and listen to patients and to validate patients’ feelings. They encouraged patients to participate in their treatment wherever possible, as this could give the patients a feeling of being in control over their own situation, while at the same time allowing the patients to give up control because they knew that they were receiving care. Sometimes, patients would make requests that were on the borderline of what is medically justifiable. In those situations, it was important to listen to the patient’s concerns and negotiate best practice.

*“When I meet the patient for the first time*,* I have about five minutes to get this patient to trust me. Then*,* I must have knowledge and preferably some experience. And I must act in such a way*,* show respect*,* kindness and care*,* so that the patient can trust me.*” (NA13).

When caring for children, it was especially important to strengthen the child’s right to self-determination by allowing the child to choose when possible and addressing the child directly, not only the parents.

*“It is about both the right and the possibility to a certain co-determination. They are in a way subject to their parents*,* so to speak*,* therefore*,* it is important that they are given the freedom to choose when possible.”* (AN15).

### Knowing oneself

The NAs reflected on their own role and what is required of a professional NA. Knowing oneself, as in being aware of one’s own strengths and limitations, affected all levels of being an NA, such as in direct contact with the patient and the ability to work safely and independently, but also in being part of the team surrounding the patient. Personal attributes, such as courage, flexibility, being solution-oriented and having the ability to handle stress, were considered central. This was also helpful when navigating ethical dilemmas and providing compassionate care in recognition of patient values and beliefs. Many situations required that they be able to think outside the box and improvise to get the job done, especially when the situation involved children or patients with intellectual disabilities, such as dementia and anxiety disorders. Some reported how they had seized the opportunity to carry out traditional nursing tasks beyond what was expected of them as an NA, such as taking care of an infected wound or giving foot care, while the patient was under general anaesthesia receiving dental treatment. It was considered important to be able to work independently and make decisions under pressure. They applied different strategies to cope with stressful situations, such as applying the ABCDE assessment tool (Airway, Breathing, Circulation, Disability and Exposure, a systematic approach used in medicine and healthcare to evaluate and manage critically ill patients) or consciously using non-technical skills. The more experience they had, the easier it became to take on a more central role on the team. Being confident in their role also meant being aware of their own limitations, being open to suggestions, asking for help, seeking a discussion partner and admitting to making mistakes.

*“It’s all about experience. It takes time to build up*,* what shall I say*,* an image-sensory database in my head*,* of clinical signs then*,* for which I don’t necessarily have such specific words.”* (NA5).

### Combining advanced knowledge with experience

Our participants emphasized the importance of a deeper understanding of the patient’s physical, psychological and social needs, as well as the ability to communicate effectively with patients, next of kin and other healthcare professionals. They relied heavily on their extended theoretical knowledge of pharmacology, surgical procedures, technology and monitoring. Furthermore, they relied on their previous clinical experience to manage complex situations while performing under pressure, making appropriate referrals and developing comprehensive care plans. They knew how to be prepared, have a backup plan in the case of an emergency and anticipate the outcome of a crisis situation. Setting priorities and working systematically was important to ensure a safe patient trajectory.

*“We measure all kinds of data that are often clear and obvious*,* such as pulse and blood pressure. But it is that feeling in addition. That you see the patient*,* this is going well or that this is a really bad patient.”* (NA9).

Some NAs also pointed out how experience comes at a price, meaning that they had experienced situations involving unwelcome incidents, often because of the team being inadequately prepared.

*“The scariest patients are often the healthy day patient who has an underlying allergy or something we don’t know about.”* (NA13).

*“It feels like getting a bucket of cold water in your face.”* (AN11).

### Aspiring towards excellence

The NAs took the lead in advancing their profession as they aspired towards excellence. This involved living up to certain expectations as a professional, exercising professional courage and seeking professional development through continuous learning.

### Living up to professional expectations

Being an NA meant that they entered a role as a professional actor, where certain expectations exist. They were expected to be well prepared and work systematically according to official clinical guidelines and standards. Handling various tasks simultaneously required seamless collaboration and situational awareness. The ability to value and acknowledge the efforts of team members facilitated prompt intervention and assistance in critical situations. It was also regarded as crucial for gaining trust among peer professionals. To take responsibility also meant fulfilling the trust that had been given to them by virtue of belonging to a particular profession. In their experience, patients normally have an expectation that the surgical team will provide and ensure their safety. Close collaboration with the anaesthesiologist contributed to increased job satisfaction, which they had not experienced to the same extent in other workplaces. They aimed to protect the patient from certain discussions and decisions by collaborating with the anaesthesiologist to create a treatment plan before the patient’s arrival, presenting the team as united and cohesive. At the same time, they would invite the patient to participate in the process when considered appropriate. This was believed to reduce patient anxiety and promote a sense of security.

*“After all*,* they surrender their bodies completely to us and lose all control.”* (NA18).

The most experienced NAs pointed out how the work culture had changed radically in recent decades, from being a hierarchical system with the surgeon at the top to focusing more on collaboration and teamwork. The use of *Safe Surgery* protocols instilled seriousness and encouraged participation. This was perceived as enhancing safety and preventing errors, especially during high-risk situations. Collaborative efforts fostered a positive synergy, as each team member would contribute to a shared goal.

“*Situational awareness is not only about getting an overview. It is also about the small and big things such as good routines for positioning the patient on the table and being focused on the entire patient.”* (NA6).

It was important to have a certain understanding of the system regarding the use of resources on behalf of the hospital and society in general. They had to be able to work efficiently without it being at the expense of the individual. Being efficient could also be perceived as caring, knowing that there are more patients waiting or even being directly beneficial to the patient, as one NA put it:

*“It somewhat matches what I’m interested in. It’s fun to be able to control the anaesthesia so that they don’t sleep for 40 minutes afterwards*,* but that you actually wake them up precisely.”* (NA2).

### Exercising professional courage

Adhering to standards also extended beyond following procedures. It involved personal judgement, seeking input from colleagues when needed and having the courage to stand up and act on behalf of the patient. The NAs were concerned about the consequences of constant demands for increased efficiency. This pressure could result in being less prepared and having insufficient time for individual adjustments. Streamlining processes often led to more people being involved in patient care, potentially compromising overall oversight and the unclear division of roles and responsibilities. The NAs feared losing control over their work situation and how this might impact effective communication and patient safety. Some reported instances of patient mix-ups during surgery, incorrect blood transfusions, medication errors and a failure to identify critical information such as allergies and underlying medical conditions. With pressure building up, they could be tempted to take shortcuts, leading to care left undone. They also considered how patients perceived interacting with numerous staff members and being asked the same questions repeatedly. This could erode patient trust in the system and create feelings of insecurity. Reflecting on the dilemma of anesthetizing nervous patients, they debated whether rapid induction was beneficial or if more time should be spent reassuring patients for a calmer awakening. They were concerned about efficiency taking precedence over human considerations, potentially leading to a loss of compassion. This challenge was particularly pronounced when administering anaesthesia to children.

A poor work environment also affected the nurses themselves. They were no strangers to handling stressful situations such as in trauma care. However, the chronic stress caused by efficiency demands significantly impacted their work environment and long-term performance. Having the courage to speak up and pump the brakes was not always easy. Many had developed strategies to multitask efficiently without compromising on quality and safety. Those who lacked the courage to raise concerns could easily find themselves unprepared in unexpected situations. In such cases, clear leadership and effective teamwork were essential.

“*We can’t afford to make any mistakes. And it is precisely in such settings that things can go wrong.*” (NA13).

### Continuously learning

There was a consistent interest in improving themselves to contribute to clinical practice development. The NAs usually lacked opportunities to receive feedback from their patients because patients are rarely seen again after being left them in a post-anaesthesia care unit. Therefore, feedback from colleagues became even more critical. Learning from each other and discussing situations with the aim of improving practice was essential. Many spoke warmly about the importance of having good role models, supporting both student and peer mentoring.

*“It’s about which supervisors you’ve had over the years*,* or which colleagues you’ve had. I can take an example from children again*,* where you see that colleagues have such a good appearance towards the child and provide you with good input on how to meet and divert children*,* and how to use the tools you have in a good way.”* (NA15).

As professionals, they valued the importance of maintaining curiosity and openness to change. The constant development of new technologies, tools and resources stimulated them towards perpetual growth and lifelong learning. The accumulation of sensory and clinical observations over time contributed to personal development. Most of the NAs emphasized not only the importance of relying on experience itself, but also that having evidence-based knowledge is equally important. As such, the recent developments in educational programmes in Norway being at a master’s degree level was emphasized as an important step. Knowing how to find and evaluate new information critically was considered crucial to improve practice and rule out things that did not work well.

*“Being able to be a little critical*,* both of what you hear and what you read. It’s a dilemma. You must be both open and curious*,* but also critical.”* (NA7).

## Discussion

Despite a long-standing history of nurse anaesthesia practice, there has been little research exploring how NAs describe their practice as being nursing when caring for surgical patients [18]. While all healthcare professionals interact with patients, NAs are characterised by their focused interaction and scope of practice. We found that NAs are grounded in their basic training as nurses through *Attending to physical and psychological needs*, *Providing a concerned presence* and *Aspiring towards excellence.* Being competent as an NA carries a dual significance: completing a formal education and obtaining a licence or authorization (*becoming* an NA), while also representing a lifelong transformative process (*being* an NA).

The data as presented in this study testify to the complexity of being an NA. Our findings correspond well with a recent meta-ethnography [11] demonstrating how NAs work at different levels to deliver fundamental holistic care, conceptualized as foregrounding (activities performed in the immediate proximity of the patient) and backgrounding anaesthetic nursing (indirect activities mostly hidden from the patient). On a foregrounding level, the NAs in our study highlighted how clinical judgement to ensure quality of care rests on a multifaceted set of skills, such as appropriate management of technical equipment, clinical procedures and safety precautions and establishing relational trust between the patient and the healthcare team. On a backgrounding level, the NAs in our study emphasized the importance of team collaboration and the ability to manoeuvre the services at a system level.

The importance of establishing a trusting relationship was a recurring topic. The surgical patient is dependent on the expertise of healthcare professionals to meet their needs. Although several validated instruments to measure and assess the presence of patients’ anxiety exists, they are not specifically related to anaesthesia and surgery, and are rarely used [25, 26]. Our informants reported that they used themselves and their previous experiences when attending to the needs of the individual. Patients’ sense of feeling safe corresponds with the ability to trust [6]. Trust in healthcare professionals involves certain expectations to those who serve them [27, 28] and has been suggested to be a potential marker for how patients evaluate the quality of care [28, 29]. In a recent qualitative review, Cheng and colleagues [5] aimed to explore surgical patients’ concerns in the perioperative period and found that a lack of trust in healthcare personnel was linked to a perceived lack of care and a breakdown in communication. Trust is perishable, and failure to instil trust may have serious consequences. Therefore, NAs have a significant ethical responsibility to uphold this trust.

Our participants were also concerned about the increased requirements for efficiency and time pressure, leaving them less prepared and sometimes unable to make individual adjustments. An unclear division of roles and responsibilities could lead to care left undone, create feelings of insecurity and was perceived as a potential threat to patient safety. Care left undone may be related to factors internal to the nurse [30] and/or work environment and quality of leadership [31]. A cross-sectional study by Marsh et al. [32] found that missed nursing care in the operating room unit was predominately reported during the preparatory phase and in relation to communication. Regardless of the underlying cause, it is important to identify contributing factors in perioperative care so that strategies and solutions can be implemented to counteract for missed care. Previous research has suggested that continuity of care should be promoted to facilitate patient participation and safety, and that preoperative information should be guided by patient expectations [26]. A recent rapid review by Leonardsen et al. [12] indicated that using a person-centred approach in delivering personalized information in an empathetic way based on the patients’ individual needs and preferences improved the quality of care. In a concept analysis of *feeling safe* perioperatively from a patient perspective, Larsson et al. [6] identified participation (e.g. to be seen as a person with individual needs), control (e.g. the ability to trust the staff) and presence (e.g. of staff or relatives) as defining attributes. This suggests the need for a conscientious and supportive management, especially in the preparatory face, to facilitate a humanized care [15, 33]. It is a paradox that invisible care only becomes visible when left undone.

The NAs in our study outlined their proactive approach to patient care, detailing how they carefully planned the patient’s care pathway well in advance, devised contingency plans and continuously assessed and tailored care to meet individual needs. This approach highlights the critical nature of a well-orchestrated nursing process pivotal for securing a reliable and safe patient care trajectory. According to Kim [34], nursing care is both concerned with the scientific problem-solving related to medical conditions and *how* to help the patient in dealing with his/her health-related situation. In addition to describing how, the NAs in our study also argued *why* when describing how clinical judgement is imperative to ensure quality of care. This requires a range of skills and attributes, including critical thinking, problem-solving, clinical reasoning and ethical decision-making, that need to be transformed into clinical practice.

The moral obligation to maintain professional standards and promote best practice was indirectly described by many as they explained how they guided students or referred to other colleagues they considered to be important role models. This may be interpreted as normative conduct because they point to what they themselves consider standards of excellence. Being grounded in nursing was an essential prerequisite for becoming and being an NA and providing the surgical patient with high-quality anaesthesia care. Further, being educated at the master’s level was highlighted as a necessity to meet future needs. The question whether advanced practice nursing educational programs need to be on a master’s level, remains controversial. However, there is growing evidence to support such a transformation, to be able to meet future needs and continue to improve the quality of health and care services [35]. Practice development involves blending different types of knowledge, experience and research into knowledge translation [36]. NAs must be able to articulate and argue for their own profession, as identified in our informants as they demonstrated professional courage. In discussing the knowledge and caring discourses in nursing theory, Bliss and colleagues [10] argue how courage and justice are connected to our capacity to be practical and listen to reason. Safety in anaesthesia practice relies on compliance to treatment guidelines and procedures, while at the same time, knowing when it is necessary to deviate from these to provide with appropriate individual care. As such, professional courage in anaesthesia nursing care is also about knowing when and arguing why.

Contextual factors pose great challenges to care in acute hospital care settings [37], and the NAs raised concerns on how chronic stress could impact their work environment and long-term performance. According to our findings, NAs need to work in an environment with supportive management. Collaborative working relationships must be based on the same principles as for good patient treatment. The management team has a formal responsibility to create a workplace that enables learning and flourishing, including creativity, innovation and the opportunity to drive quality improvement and knowledge development [38]. Failure to do so may result in fatigue and burnout, and potentially have a direct impact on patient safety [39]. Staff shortages and time restrictions may lead to other team members taking over central nursing tasks or, at worst, care left undone.

### Methodological considerations

The *Consolidated Criteria for Reporting Qualitative Research Checklist* [40] was used in the preparation of the manuscript with the aim of providing the reviewers with sufficient details to assess the rigour of the analysis and the credibility of the findings [41]. In qualitative content analysis, trustworthiness depends on the logic trail of the three main phases of preparation, organization and reporting of the results [42], leaving it up to the reader to be the final judge. This study has a strength in its broad sampling collection. In the methods- and results-section, we have provided the reader with sufficient descriptions of the context, selection and characteristics of the participants. In terms of judging the quality of the data, we prefer to use the term *guided by information power* as suggested by Malterud [43]. We found that the interviews were characterized by strong dialogue, providing us with rich data within a narrow aim of interest. The findings are supported by quotations from the informants.

Qualitative content analysis is not a linear process; rather, we moved back and forth, shifting between decontextualization and recontextualization [23], discussing and revising the analysis. We had several reflective and critical discussions, taking our own preunderstanding into consideration, before finally agreeing on the findings. In qualitative research, reflexivity is paramount [44]. Despite the inductive approach, the authors’ professional backgrounds (anaesthesia and intensive care nursing) are likely to have influenced our interpretations, both as a strength and a limitation. All raw data were retrieved from a Norwegian context. This needs to be taken into consideration in terms of validity and transferability to other countries and clinical settings.

## Conclusion

Patients should be able to depend on the ability of NAs to provide them with not only safe, but also individual care. With their foundational education in nursing and extensive training in advanced anaesthesia nursing practice, NAs perceive themselves as being highly qualified to address the needs of surgical patients in vulnerable situations. Their nursing background provides them with a distinctive understanding of the existential concerns faced by patients and has prepared them to respond accordingly. As part of the surgical team, they are uniquely positioned to promote continuity of care and address any potential gaps in care that may arise, identified in this study as knowing how and arguing why. Exercising professional courage aids in upholding standards of excellence and makes the invisible explicit. Limited resources in healthcare are a reality. Therefore, the content of nursing in anaesthesia must be expedient and meet future needs. Being an NA encompasses a desire for lifelong learning driven by internal motivation but must be facilitated and supported by external factors. Knowledge from this study may be applied to gain a better understanding of NAs’ scope of practice and perception of nursing, adding to the field of describing and understanding advanced nurse anaesthesia practice in the perioperative context and supporting supervision in postgraduate education. Given that education and legislation within anaesthesia practice differs globally, information from this study may be useful in informing health policies and governmental regulations, with the goal of improving education, quality of anaesthesia care and patient safety. Future research should include observational studies to identify opportunities for nursing in anaesthesia care and how they are used, including analysing barriers and facilitators to identify specific areas for improvement in anaesthesia nursing practice.

## Data Availability

The datasets used and/or analysed are available from the corresponding author upon reasonable request.

## Declarations

### Authors’ information

Aina L. Bjerkeli Lekens, RNA, MScN, PhD candidate.

Sigrunn Drageset, RNA, PhD, Associate Professor.

Britt Sætre Hansen, CCN, PhD, Professor.

## Abbreviations

NA: Nurse anaesthetist
APN: Advanced practice nurse
ICN: International council of nurses
IFNA: International federation of nurse anaesthetists
